# The effect of mobility reductions on infection growth is quadratic in many cases

**DOI:** 10.1038/s41598-024-64230-1

**Published:** 2024-06-24

**Authors:** Sydney Paltra, Inan Bostanci, Kai Nagel

**Affiliations:** 1 Technische Universität Berlin, FG Verkehrssystemplanung und Verkehrstelematik, 10623 Berlin, Germany; 2 Zuse Institute Berlin, 14195 Berlin, Germany

**Keywords:** Infectious diseases, Applied mathematics

## Abstract

Stay-at-home orders were introduced in many countries during the COVID-19 pandemic, limiting the time people spent outside their home and the attendance of gatherings. In this study, we argue from a theoretical model that in many cases the effect of such stay-at-home orders on incidence growth should be quadratic, and that this statement should also hold beyond COVID-19. That is, a reduction of the out-of-home duration to, say, 70% of its original value should reduce incidence growth and thus the effective R-value to $70\% \cdot 70\% = 49\%$ of its original value. We then show that this hypothesis can be substantiated from data acquired during the COVID-19 pandemic by using a multiple regression model to fit a combination of the quadratic out-of-home duration and temperature to the COVID-19 growth multiplier. We finally demonstrate that many other models, when brought to the same scale, give similar reductions of the effective R-value, but that none of these models extend plausibly to an out-of-home duration of zero.

## Introduction

### Motivation

The COVID-19 pandemic confronted nations worldwide with a serious medical crisis, but also a cascade of economic, social, and psychological challenges. In this context, many countries implemented non-pharmaceutical interventions (NPIs^1^), initially as part of a suppression strategy, but later as part of a risk mitigation strategy. Throughout the implementation of these NPIs, and particularly in the aftermath, significant efforts have been made to quantify their effectiveness and their interrelation with disease indicators, such as the effective *R*-value and hospitalization numbers.

For example, an early work by^2^ focuses on the first wave of the pandemic to link NPI introduction dates to national case and death counts. The authors find that school and university closures are most effective in reducing transmissions. However, their approach cannot distinguish direct effects on transmission within these institutions from potential indirect effects, such as changes in public behavior prompted by the signaling effect of school closures. Furthermore, they note that the disease burden may have been reduced by unobserved NPIs or voluntary behavior (e.g. mask wearing). In a follow-up study by the same group^3^, a significantly smaller effect size of the NPIs was observed during the second wave compared to the first. They attribute this decrease to differences in pre-intervention contact patterns, safety measures, personal protective behaviors, and the reduced adherence to NPIs. Notably, the impact of school closures was much smaller during the second wave, possibly influenced by safety measures and behavioral changes (e.g., symptom screenings, asymptomatic testing, sanitizing) at schools. The authors speculate that the timing of school closures during the second wave, as they were now among the later NPIs, may have contributed to a reduced signaling effect.

Another example is the work by^4^ that analyzed NPIs implemented in March-April 2020 across various countries. The study reveals that canceling small gatherings had the most significant impact on the effective R-value, followed by closing educational institutions. They find that for the closure of educational institutions and the cancellation of small gatherings, among many other NPIs, an early implementation is always more beneficial. The study concludes that NPI effectiveness is influenced by governance, epidemic stage, socio-economic factors, cultural and political characteristics, and previously implemented NPIs.

In the three aforementioned works, NPIs are directly translated to disease indicators. During their study period, namely the first wave of the pandemic, NPIs were only implemented over a couple of weeks. However, this changed from the fall of 2020 onwards, when NPIs (at least in western European countries) were implemented over longer periods of time. This duration of implementation necessitates the consideration of an intermediate translation step: time-dependent compliance as discussed in more recent works by^5–8^. Consequently, we decompose the effect of government-mandated NPIs into two steps:

1. Government-introduced NPIs influence the population’s behavior.
2. The population’s behavior influences the (effective) R-value/incidence growth.

In this work, we concentrate on the second step. That is, we do not discuss how strictly the population follows government-mandated behavior, and instead investigate how revealed behavior, by which we mean the sum of voluntary and state-mandated behavior as well as seasonal behavior adaptations, influences incidence growth. Here, the availability of mobility data from cellphones serves as a proxy for revealed behavior, thus allowing investigations hitherto impossible. Indeed, many studies have stressed correlation and plausible causal connection between mobility and incidence growth; see “Relationship between COVID-19 and mobility world-wide” section for details. In this paper, we go beyond these in the following way: First, we propose a theoretically motivated linear-in-parameters regression model, which we test with empirical data, and use to investigate mobility’s potential to serve as a proxy for additional self-protective behavior like mask-wearing. Then, we translate models from related work into our proposed model and show thereby that the effect size we found is consistent with the literature.

One important outcome of our investigations is that the effect of mobility reductions in many situations is *quadratic*. That is, a reduction of the population mobility to, say, $70\%$ of its normal value leads to a reduction of incidence growth to $70\% \times 70\% = 49\%$ of its original value.

We begin by reviewing the currently existing literature, presenting alternative models and their results on the impact of mobility on disease spread. In the methods section, we motivate the quadratic effect of mobility reductions theoretically before introducing the variables of interest and presenting an exploratory analysis. In the results section, we first investigate multiple mobility-only models, settling on the quadratic no-intercept version, before arguing for the inclusion of a temperature-related variable and deriving our final regression model. We then discuss the implications of the chosen model, and in particular compare it to other models.

### Literature review

#### Relationship between COVID-19 and mobility in Germany

Multiple studies investigate the reduction of mobility in Germany related to the COVID-19 pandemic. As none of these studies quantitatively investigates the consequences of these reductions for the infection dynamics, they are summarized in Supplementary section 6.2.

#### Relationship between COVID-19 and mobility world-wide

Taking on a global perspective, we discuss studies which quantify the influence of mobility reductions on the infection dynamics. For the first four models described in the following, we have been able to reconstruct the full model. Thus, we present them in more detail and compare them to our model in “Comparison of our model to models in the literature” section.

Nouvellet et al.^9^ use mobility data as a proxy measure for social distancing to characterize the relationship between transmission and mobility for 52 countries during the first year of the pandemic. They split the year into two country-specific periods, estimating for each period the log of the effective reproduction number as a function of the log of the basic reproduction number and a mobility indicator. The authors compute an adjusted $R^2$ of 0.94 for the 1st period (before the change of the relationship occurred) and an adjusted $R^2$ of 0.45 for the second period (after the change of the relationship occurred).

Considering a similar time frame and using the mobility indicators from the Google mobility reports for 125 countries as well as 52 US states/regions, the authors of^10^ analyze the correlation between effective reproduction number and mobility. Using Pearson correlation test and linear modeling, the authors identify countries for which the correlation between $R_t$ and mobility indicators was either (a) negative, (b) positive, or (c) more complex than a linear relationship. In case of (c), they additionally presented a quadratic model to improve their results.

Noland^11^ uses a fixed-effects model that controls for state-level effects to explore the relationship between Google mobility data and $R_t$ (considering a 7-day and 14-day lag) on US state level, finding a positive correlation for retail/recreational activity and public transit, smaller association for shopping at grocery stores and pharmacies, and negative coefficients for time spent in residential areas.

Concentrating on five public health units (PHU) in the Greater Toronto Area in Canada and with the help of segmented regression^12^, Dainton and Hay assess the relationship between the effective R-value $R_t$ and each Google mobility variable separately. They and find a more pronounced relationship during the first wave than during the second wave in Canada.

Using mobile phone data from Teralytics^13^, Badr et al. demonstrate how the relative change in mobility (as a proxy for social distancing) correlates with the rate of new infections in the 25 US counties that had the highest number of confirmed cases on April 26, 2020. The authors fit a generalized linear model for each county, using a generalized Covid-19 growth rate, defined as the logarithm of the average number of new cases over the previous 3 days divided by the logarithm of the average number of new cases over the previous 7 days. The large divergence between this approach and the methodology proposed in this paper does not allow for a meaningful comparison, and is therefore omitted from our discussion in “Comparison of our model to models in the literature” section.

Finally^14–20^, find correlations between mobility and COVID-19 indicators, but do not present coefficients that may be compared to our model^14^: introduce an SIR model, whose parameters depend on social restrictions. These parameters are estimated by fitting the model to national death data, and the authors discuss restrictions’, which include mobility reductions’, ability and duration to reduce the R-value to one.^15^ present a simple statistical model using mobility data, day of-week variables, and indicators for changes in testing regimes that allows the generation of a 10-day forecast for 80 countries.^17^ find that travel between US counties decreased by up to 35% during the first wave, but recovered rapidly during the partial reopening phase, and^16^ showed with the help of an SEIRL (where L stands for isoLated) model that initial lockdowns and mobility suppressed the first COVID-19 wave in 12 global regions. Focusing on data from the first year of the pandemic^20^, Nanda et al. relate all Google mobility categories apart from “residential” to new daily confirmed cases, finding that different kinds of community mobility were significant predictors of COVID-19, while the authors of^19^ relate population, temperature, mask compliance, and the first principal component of Google’s six mobility variables to the log of the weekly infection growth rate and identify considerable spatiotemporal variations. Considering the first Omicron surge in the US^18^, Harris uses principal component analysis to relate Google mobility to COVID-19 case incidences in US counties, finding the coefficient of the “residential” mobility category to be the second largest in magnitude and the only one that is negative (PCA coefficient $= -0.4571$).

## Methods

### Theory: relating mobility to the growth multiplier

In our previous work^21^, we discuss how the reduction of participation has a *quadratic* effect for gatherings. Work by^22^, which focuses on the special case of in-person work meetings, comes to the same conclusion: If only a fraction $\alpha < 1$ of a base turnout is present, then only that fraction $\alpha$ can bring the infection to the gathering, just as only a fraction $\alpha$ can become infected. In consequence, the base number of infections is multiplied by $\alpha \cdot \alpha = \alpha ^2$ under such circumstances.

More precisely, for a gathering of *N* persons, where $p_{contag}$ denotes the probability to be contagious, $p_{contag} \cdot N$ is the expected number of contagious attendees. On the other hand, when $p_{ inf}$ denotes the probability to get infected (conditional on being in the same room with an infected person), then the expected number of attendees who get infected per contagious person is $p_{ inf} \cdot (N-p_{contag} \cdot N)$, i.e. the overall expected number of infected persons is

$$\begin{aligned} {\mathbb {E}}[I_{ fullTurnout}] = p_{contag} \cdot N \cdot p_{ inf} \cdot (N-p_{contag} \cdot N) \ . \end{aligned}$$

If the turnout is reduced by multiplying it with a factor $\alpha < 1$, the expected number of infections is reduced to

1 $$\begin{aligned} {\mathbb {E}}[I_{reducedTurnout}]&= p_{contag} \cdot \alpha N \cdot p_{ inf} \cdot ( \alpha N - p_{contag} \cdot \alpha N ) \nonumber \\&= \alpha ^2 \cdot \Big ( p_{contag} \cdot N \cdot p_{ inf} \cdot ( N - p_{contag} \cdot N ) \Big ) \nonumber \\&= \alpha ^2 \cdot {\mathbb {E}}[I_{ fullTurnout}] \ . \end{aligned}$$

To illustrate this, let us consider an example: Assume 101 persons, all living in single-person households, and all working for multiple hours every day together in an open-plan office. Now assume that one of these persons gets infected and then contagious, and then carries that infection into the office. Let us also assume that this contagious person develops symptoms after two contagious days and from then on stays home.

If the contagious person is at the office, then he/she emits virus particles into the air. For COVID-19, we can assume in leading order that the virus particles are uniformly mixed into the air volume, exposing all the other 100 workers to a certain virus dose *d*. (We discuss other infection mechanisms later in the paper.) Because the air volume of the open plan office is large, the resulting concentration and thus the inhaled dose are small, meaning we are in the virus-limited regime^23^, and the probability to get infected, $p_{inf}$, is small and approximately proportional to the dose:

$$\begin{aligned} p_{inf} = 1 - \exp (-\Theta \cdot d) {\mathop {\longrightarrow }\limits ^{d \rightarrow 0}} \Theta \cdot d \ . \end{aligned}$$

Since all 100 persons are exposed to this dose, the expected value of secondary infections is

2 $$\begin{aligned} {\mathbb {E}}[I_{fullTurnout}] = 1 \cdot p_{inf} \cdot 100 \cdot 2 \ , \end{aligned}$$

where the factor of “2” takes into account that this happens over two consecutive days.

Now assume that out of the susceptible 100 persons, only 50 randomly selected persons are coming into the office. The resulting expected value of infections is

3 $$\begin{aligned} p_{inf} \cdot 50 \cdot 2 = \frac{{\mathbb {E}}[I_{fullTurnout}]}{2} \ , \end{aligned}$$

i.e. *half* of Eq. (2). Note that, in the virus-limited regime, the result does not depend on the question if the same or different 50 persons are at the office on the second day, since the probability of not being able to catch an infection on the second day because one already caught it on the first day is small.

Now assume that the contagious person also follows the reduced office attendance. There are four cases, all with equal probability of 1/4:

1. The contagious person is at the office on both days. The resulting expected value of secondary infections is Eq. (3), i.e. ${\mathbb {E}}[I_{fullTurnout}]/2$.
2. The contagious person is at the office on the first but not the second day. The resulting expected value of secondary infections is *half* of Eq. (3), i.e. ${\mathbb {E}}[I_{fullTurnout}]/4$.
3. The contagious person is at the office on the second but not on the first day. The resulting expected value of secondary infections is again *half* of Eq. (3), i.e. ${\mathbb {E}}[I_{fullTurnout}]/4$.
4. The contagious person is at the office on neither of the two days. The resulting expected value of secondary infections is zero.

The expected number of secondary cases reads

$$\begin{aligned} {\mathbb {E}}[I_{reducedTurnout}]&= \frac{1}{4} \cdot \frac{{\mathbb {E}}[I_{fullTurnout}]}{2} + \frac{1}{4} \cdot \frac{{\mathbb {E}}[I_{fullTurnout}]}{4} + \frac{1}{4} \cdot \frac{{\mathbb {E}}[I_{fullTurnout}]}{4} + \frac{1}{4} \cdot 0 \\&= \frac{{\mathbb {E}}[I_{fullTurnout}]}{4} \ . \end{aligned}$$

That is, the expected value of secondary infections is 1/4 of the original value, achieved by reducing office attendance by 1/2. Replacing the reduction to 1/2 by $\alpha$ leads us to Eq. (1). Complementary to the theoretical explanation, simulation results for $\alpha \in [0.1,1]$ can be found in Supplementary Section 6.1.

The above approximation breaks down once we leave the linear regime of small virus doses and therefore small infection probabilities. Furthermore, in order to transfer the quadratic effect of attendance reduction of a single event to overall mobility reductions (in this work described via out-of-home duration), it is also necessary that all activity types (e.g. leisure, school, work) are reduced (roughly) equally. Assume, for example, a situation in which only two out-of-home activities A and B are available, which have exactly the same characteristics (e.g. contact intensity, room size, indoor/outdoor activity, duration). Reducing participation at both to 50% yields a reduction of the out-of-home duration by half and a quadratic reduction of infections to $50\% \cdot 50\% = 25\%$. Conversely, completely shutting down activity A while leaving activity B completely open, also reduces the out-of-home duration (in average) by half but reduces infections only by $50\%$. On the contrary, no assumptions about the reasons of the mobility adaptations need to be made and no differentiation between mandated, voluntary, or seasonal behavior adaptations is necessary.

In consequence, both linear and quadratic dependencies of the growth multiplier on out-of-home duration are consistent with theory, making the type of relationship dependent on a country’s handling of the pandemic. Furthermore, even higher order terms could be possible when the reduction of the out-of-home duration correlates with other infection-suppressing behaviors such as mask compliance or deliberately moving gatherings outdoors despite cold weather. In this case, we would observe the effect of reduced out-of-home duration, multiplied with the additional effects for which out-of-home duration would be a proxy. The following sections empirically test the just-introduced theory for Germany: The relation of the time-dependent and thus varying out-of-home duration (in other words different values for $\alpha$) and the time-dependent and thus varying COVID-19 growth-multiplier (in others words different values for expected no. of infections) is explored.

### Empirical methods

#### Variables

Our considered response variable is:

- ${\textbf {G}}_t {:}{=}\frac{I_{t+1}}{I_t}$, the multiplicative growth rate, which describes the change of incidence from one week to another. Hereby, $I_t$ is the weekly average 7-day incidence per 100,000 at time *t*. Incidence data is taken from the RKI^24^, which provides the German national 7-day-incidence/100,000 on a daily level.

The considered explanatory variables are:

- ${\textbf {D}}_t$: “Out of home duration”, which serves as a mobility indicator. Denotes in hours how much time an average person spends daily outside their home during week *t*. The daily out-of-home duration on the postal code level is provided by the commercial company Senozon. Our research group has aggregated the data on a national level and weekly level. The aggregated data can be accessed via Zenodo^25^.
- **Tmax**$_t$: Weekly average of the daily maximum temperature (in Celsius), see “Outdoor fraction” section for details. We used publicly available data provided by Meteostat^26^.
- **Tavg**$_t$: Weekly average of the daily average temperature (in Celsius), see “Outdoor fraction” section for details, and find the raw data on Meteostat^26^.
- **Precip**$_t$: Weekly average of daily total precipitation (in mm), and find the raw data on Meteostat^26^.
- **outFrac**$_t$: “Outdoor fraction”, a transformation of ${\textbf {Tmax}}_t$ following the example of^21^ and computed in two steps: This approach is motivated by the argument that, if the effect of the temperature is mostly transmitted via the indoors/outdoors behavior, it needs to saturate at the lower end because eventually all activities are indoors, and at the upper end because eventually all activities are outdoors. Such a behavior is typically modeled with an S-shaped function such as a logit function. Those functions are characterized by a midpoint and a slope at that midpoint. Thus, first, we introduce a time-dependent midpoint $T^{\star }_t$ equal to
  $$\begin{aligned} T^{\star }_t&= {\left\{ \begin{array}{ll} 7.5/30 * t + 17.5 &{} \text{ if } t \le 30, \\ 25 &{}\text { if } t > 30. \end{array}\right. } \end{aligned}$$
  In the second step, we then approximate said S-shaped function with a piecewise linear function with midpoint $T^*_t$ and slope 0.1:
  $$\begin{aligned} {\textbf {outFrac}}_t({\textbf {Tmax}}_t)&= {\left\{ \begin{array}{ll} 0 &{}\text { if } {\textbf {Tmax}}_t < T^{\star }_t -5, \\ \frac{{\textbf {Tmax}}_{t} - (T^{\star }_t-5)}{10} &{}\text { if } T^{\star }_t - 5 \le {\textbf {Tmax}}_t \le T^{\star }_t + 5, \\ 1 &{}\text { if } {\textbf {Tmax}}_t > T^{\star }_t + 5, \end{array}\right. } \end{aligned}$$
  where ${\textbf {Tmax}}_t$ denotes the weekly average maximum temperature (in Celsius). This approach is motivated by our modelling work in fall 2020, where a higher sensitivity to sinking temperatures after a warm summer was a plausible explanation for the timing of the beginning of the second wave^21^. Hence, at $t=1$ (corresponding to “15/March/2020”), people are willing to spend half of their time outside if $T^{\star } = 17.5$. Over the course of the year, this threshold linearly increases until it reaches $T^{\star } = 25$ in the fall at $t=30$ (corresponding to “04/October/2020”).

#### Time scale and lag

We consider data of the first year of the pandemic, ranging from March 15, 2020 until December 13, 2020. Because the incidence in Germany was fluctuating strongly in February and early March 2020 due to small numbers and issues with the reporting system, February 2020 is excluded. As the German government announced stricter measures on Dec 13, 2020, which were installed on Dec 16, 2020^27^, the mobility data between December 13 and December 16 was heavily influenced by this announcement. Furthermore, testing and reporting during Christmas was delayed and unreliable, leading us to the exclusion of mobility data from December 13, 2020 onwards. Time is given in weeks, where $t=1$ corresponds to “15/Mar/2020” and the maximum $t=40$ corresponds to “13/Dec/2020”. Since the growth rate is defined as $G_t = I_{t+1}/I_t$ (see above), the considered lag effectively is 2.5 weeks. Discussion of a lag of (and consequently rejection) of 0, 1, 3, and 4 weeks can be found in the Supplementary Material.

#### Exploratory analysis

##### Growth multiplier

**Figure 1:**
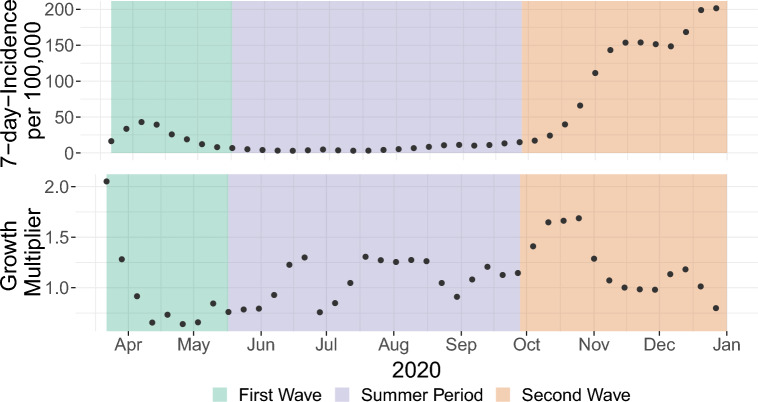
Top: 7-day-incidence/100,000 over the course of 2020. Bottom: Growth Multiplier for 2020.

The top plot of Fig. 1 shows the weekly average of the reported 7-day incidence/100,000 as provided by Robert-Koch-Institut (RKI)^24^. In Germany, the first COVID-19 wave began in calendar week 10 (starting 02/March/2020) and lasted until calendar week 20 (ending on 17/May/2020). It was followed by a summer period of low infection numbers, before the second wave began on 28/September/2020^28^. The bottom plot depicts the multiplicative rate by which the COVID-19 case incidence changed from week to week (see “Variables” section for definition).

##### Out of home duration

**Figure 2:**
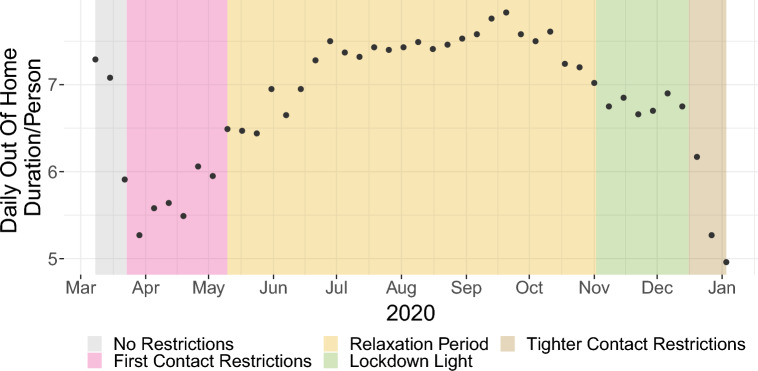
Daily out of home duration per person (in hours) for 2020.

Figure 2 depicts the amount of time (in hours) an average person spends outside their home per day. We note that when contact restrictions were introduced (23/March/2020), the out-of-home duration had already decreased and it reached its minimum in the week of the introduction of the contact restrictions. The out-of-home duration did not plateau at this minimum and in consequence, an increase can already be seen before the first NPIs were officially relaxed in calendar week 19/20 (beginning on 04/May/2020 and 11/May/2020 respectively)^29^. During the summer, out-of-home duration surpassed the pre-pandemic level of early March 2020, which is expected when comparing summer and early spring duration. Finally, starting in late September we see another decrease in out-of-home duration. During the so-called “lockdown light”, which was introduced on 02/November/2020, we observe an additional local maximum (potentially due to hasty Christmas-shopping) before the introduction of more restrictive contact restrictions on 16/December/2020.

##### Outdoor fraction

**Figure 3:**
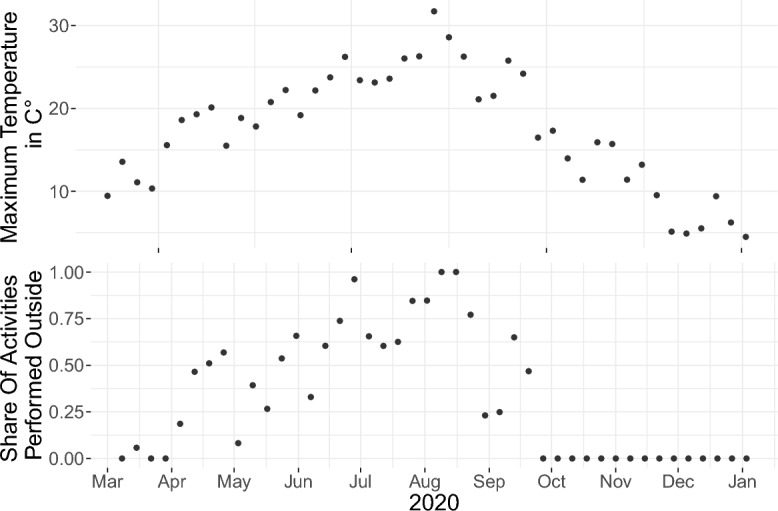
Top: Maximum temperature (in C°). Bottom: Share of leisure activities spent outside.

The top plot of Fig. 3 depicts the maximum temperature (in C°) over the course of 2020. We first average over weeks, then over federal states. For the latter, we take the weekly average of the maximum temperature of every federal state’s capital. We then average over these 16 values, weighting values according to states’ population shares. Bottom plot: As explained in “Variables” section, we assume that there exists a season-dependent threshold below (or above) which the share of leisure activities performed inside (or outside) does not further decrease (or increase). In consequence, the maximum temperature is transformed to the so-called “share of activities performed outside”, or in short “outdoor fraction”.

## Results

In this section, we present our findings on how mobility affects disease spread. That is, we use the different weekly out-of-home durations as exogeneous variable, representing different values of the reduced turnout $\alpha$ of Eq. (1). We then use the growth multiplier, explained above, delayed by effectively 2.5 weeks as dependent variable. Our model comparison confirms that mobility’s influence in Germany was indeed quadratic. Moreover, including a weather variable improves the result, but mobility has a stronger influence than weather. Notably, weather only shows an effect in models that include mobility, underscoring the intricate interplay between these variables in shaping disease transmission dynamics.

**Table 1:** Model variables: For details and data sources see “Variables” section.

Variable	
G	Growth multiplier, defined as ${\textbf {G}}_t = I_{t+1}/I_t$, describes the change of incidence from one week to another
D	Out-of-home duration, mobility variable that denotes how much time (in hours) an average person spends outside their home
outFrac	Outdoor fraction, transformation of temperature, describes the fraction of leisure activities a person performs outside

### Mobility versus weather

**Figure 4:**
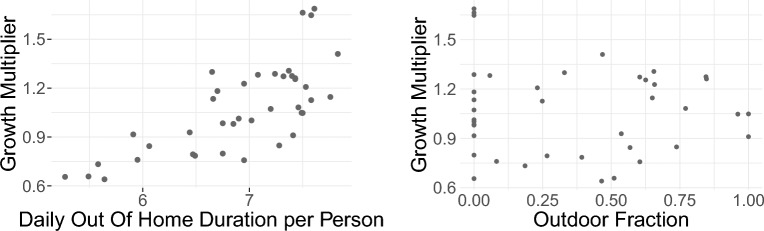
Left: growth multiplier **G** vs. out-of-home duration (in hours) **D**. Note that a base out-of-home duration of approximately 8 h corresponds to a growth multiplier of around 1.5. Right: growth multiplier **G** vs. outdoor fraction **outFrac**. Growth multiplier lags behind out-of-home duration and outdoor fraction by 2 weeks.

The left panel of Fig. 4 implies a correlation between out-of-home duration ${\textbf {D}}$ and the growth multiplier, defined as $G_t = I_{t+1}/I_{t}$, where *I* is the incidence of new cases and the index *t* numbers the weeks (see Table 1 and “Variables” section for details). In contrast, there is no obvious correlation between the outdoor fraction and the growth multiplier (Fig. 4, right panel), where the outdoor fraction is a transformation of the temperature taking into account that for very low and very high temperatures there needs to be saturation of the influence of weather; again see “Variables” section for details. We thus start with a model based on the out-of-home duration before introducing the outdoor fraction. Because of the time lag between exposition to the virus and the reporting of cases, we employ a lead of effectively 2.5 weeks on the reported case numbers (the detailed Model Comparison can be found in Supplementary Section 6.5).

### Mobility only models

We now compare multiple mobility only models to establish if the influence of mobility on disease spread is linear, quadratic or cubic:

4 $$\begin{aligned} {\textbf {G}}_{t+2} = \beta _0 + \beta _{d} {\textbf {D}}_t, \end{aligned}$$

5 $$\begin{aligned} {\textbf {G}}_{t+2} = \beta _0 + \beta _{d^2} {\textbf {D}}^2_t, \end{aligned}$$

6 $$\begin{aligned} {\textbf {G}}_{t+2} = \beta _0 + \beta _{d^3} {\textbf {D}}^3_t, \end{aligned}$$

where ${\textbf {D}}$ is the out-of-home duration in hours. The coefficients, which are computed using ordinary least squares, and summary statistics can be found in Table 2: The three models show similar values for $R^2$, the Akaike Information Criterion (AIC), and the Bayesian Information Criterion (BIC), and cannot be differentiated on these criteria alone.

**Table 2:** Regression output for different model types: linear, quadratic, cubic, quadratic without intercept, and logarithmic.

Model	Adj. $\mathbf {R^2}$	AIC	BIC	Variable	Coefficient	Std. error	${p > |t|}$
Linear (4)	0.5207	− 16.45	− 11.38	Intercept	− 0.9384	0.3068	0.0041
				**D**	0.2910	0.0442	8.98e−08
Quadratic (5)	0.5374	− 16.82	− 11.75	Intercept	0.0105	0.1626	0.949
				**D**^2^	0.0220	0.0033	7.49e−08
Cubic (6)	0.527	− 16.97	− 11.90	Intercept	0.3278	0.1156	0.0073
				**D**^2^	0.0022	0.0003	6.97e−08
Quadratic w/o intercept (7)	0.525	− 18.82	− 15.44	Intercept	0	n/a	n/a
				**D**^2^	0.0022	0.0006	<2e−16
Logarithmic (8)	0.589	− 27.41	− 22.34	Intercept	− 3.6382	0.4881	6.09e−09
				log(**D**)	1.9064	0.2528	4.61e−09

The out-of-home duration **D** leads by 2.5 weeks.

**Figure 5:**
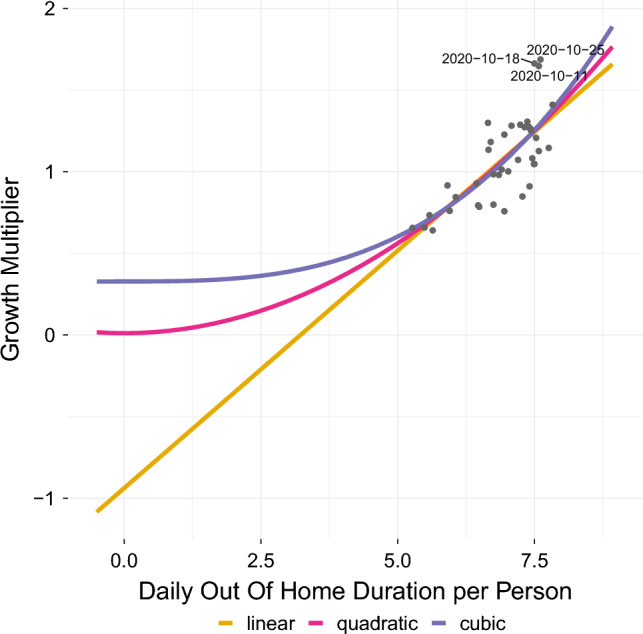
Growth multiplier vs. (average) daily out-of-home duration per person (in hours). Depicted are the regression lines of the linear (4), quadratic (5), and cubic (6) model.

In this situation, our theoretical framework coincides with (5): For an out-of-home duration **D** close to zero, meaning that persons spend little time outside their own home, we would expect almost no disease spread, or in other words, a growth multiplier close to zero. According to Table 2, only the quadratic model has an intercept not significantly different from zero. The underlying reason for models (4) and (6) to compute an intercept significantly different from zero is that data on out-of-home duration ${\textbf {D}}$ during the pandemic in Germany ranges only from 5 to 9 h per day (see Fig. 5). Hence, a linear model would need to start from an intercept of almost $-1$ to account for the relatively large slope of the data points within the observed range. Likewise, a cubic model would necessitate an intercept above zero to avoid an overly steep slope within the observed range.

Estimating the quadratic model (5) without the intercept, after inserting the estimated values (cf. Table 2), leads to

7 $$\begin{aligned} \boxed {\begin{aligned} {\textbf {G}}_{t+2} = 0.022 \cdot {\textbf {D}}_2^2 \ . \end{aligned}} \end{aligned}$$

To further confirm an exponent of 2, we estimate the exponent rather than just testing it for various integer values. We undertake this with linear regression by log-transforming the equation

8 $$\begin{aligned} {\textbf {G}}_{t+2}&= \gamma _0 {\textbf {D}}_t^{\gamma _d} \end{aligned}$$

9 $$\begin{aligned} \Leftrightarrow \log ({\textbf {G}}_{t+2})&= \log (\gamma _0) + \gamma _d \log ({\textbf {D}}_t) \nonumber \\&= {\tilde{\gamma }}_0 + \gamma _{d} \log ({\textbf {D}}_t), \end{aligned}$$

where we introduce $\tilde{\gamma }_0 {:}{=}log(\gamma _0)$. The regression output of (9) can be found in Table 2. Translated back into (8), this means

10 $$\begin{aligned} \boxed {\begin{aligned} {\textbf {G}}_{t+2} = 0.0263 \cdot {\textbf {D}}_t^{1.9064}. \end{aligned}} \end{aligned}$$

This is consistent with the quadratic model (7). In consequence, we continue with the quadratic no-intercept model.

### Models including outdoor fraction

Using mobility alone, we are able to explain more than half of the variance in the growth multiplier. It is well known that seasonality also influences COVID-19’s infection dynamics^30–32^. There are multiple mechanisms that may play a role, including that the stability of the virus may depend on ambient temperature, ambient humidity, or UV radiation, which is higher during summer. Here, we explore incorporating the outdoor fraction **outFrac** in addition to and as a cross term with mobility into our analysis. This yields

11 $$\begin{aligned} {\textbf {G}}_{t+2} = \beta _{d^2} {\textbf {D}}_t^2 +\beta _o {\textbf {outFrac}}_t + \beta _{od^2} {\textbf {D}}_t^2 \cdot {\textbf {outFrac}}_t. \end{aligned}$$

The regression output is given in Table 3.

**Table 3:** Regression output for different modes including the outdoors fraction.

Model	Adj. ${R^2}$	AIC	BIC	Variable	Coefficient	Std. error	${p > |t|}$
(11)	0.6345	− 25.2865	− 18.5310	${\textbf {D}}^2$	0.0242	0.0008	<2e−16
				**outFrac**	0.0167	0.3976	0.967
				${\textbf {D}}^2 \cdot {\textbf {outFrac}}$	− 0.0053	0.0074	0.478
(12)	0.6345	− 27.2846	− 22.2180	${\textbf {D}}^2$	0.0242	0.0008	< 2e−16
				${\textbf {D}}^2 \cdot {\textbf {outFrac}}$	− 0.0050	0.0015	0.0017

The out-of-home duration **D** leads by 2.5 weeks.

Two reasons speak against including the linear term ${\textbf {outFrac}}$. The first reason is a statistical one: Its coefficient is not significant and displays the highest p-value (see Table 3). The second reason is based on theory: Without mobility, the outdoor fraction has little to no influence on the infection dynamics. If a primary case is brought into a household, which spends all its time inside, then the whole household may become infected. But, if the household isolates itself, then no secondary cases outside the household will occur and the primary case will not influence society’s growth multiplier. Consequently, **outFrac** is dropped and we consider the model

12 $$\begin{aligned} {\textbf {G}}_{t+2} = \beta _{d^2} {\textbf {D}}_t^2 + \beta _{od^2} {\textbf {D}}_t^2 \cdot {\textbf {outFrac}}_t, \end{aligned}$$

for which Table 3 shows the estimation results, with no discernible loss in explanatory power, improved AIC and BIC, and now all coefficients significant. With this, the overall mobility effect of 0.022 from Model (7) is split into a base positive effect of 0.024 and a negative effect of $-0.005$ stemming from the interaction of mobility and the outdoor fraction:

13 $$\begin{aligned} \boxed { \begin{aligned} {\textbf {G}}_{t+2}&= 0.024 \cdot {\textbf {D}}_t^2 - 0.0050 \cdot {\textbf {D}}_t^2 \cdot {\textbf {outFrac}}_t \\&= (0.024 - 0.0050 \cdot {\textbf {outFrac}}_t) \cdot {\textbf {D}}_t^2 \ . \end{aligned} }\end{aligned}$$

### Final model

The model (13) is supported by theory, and it also displays the best fit of all models considered so far. In Supplementary Section 6.5, we consider an exhaustive comparison between model variations with terms up to the third degree, and arrive at the same result. We therefore keep (13) as our final model and inspect it further in this section.

**Figure 6:**
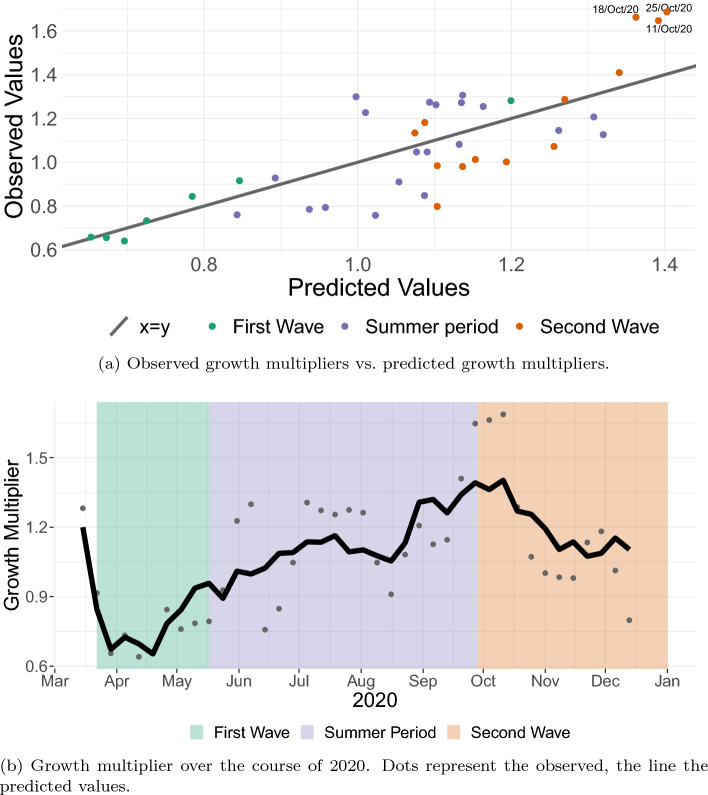
Top: Observed growth multipliers vs. predicted growth multipliers. Bottom: Growth multiplier over the course of 2020. Dots represent the observed, the line the predicted values.

Figure 6a displays the predicted vs. the observed growth multiplier in different colors for the first wave, the summer period, and the second wave for the final model (13). From the figure, we infer that the final model (13) is able to fit the data well. During the first wave, apart from the earliest data point, the observed as well as the predicted values are smaller than 1 – the reason is that the lagged growth data starts at the end of March, when the peak of the first wave had almost been reached (see Fig. 1). Then, over the summer, the observed values display strong fluctuations, taking on values between $\sim \!\! 0.75$ and $\sim \!\! 1.35$, where our fit predicts values closer to 1. This is consistent with a low incidence situation, where the “true” value is actually close to one, but random effects cause strong relative fluctuations. During the second wave, the three largest values are observed (see top-right corner of the plot) but the model underestimates the infection growth. Figure 6b displays the observed and the predicted growth rate over time. The plot over time again underlines one shortcoming of the least squares method: When strong relative fluctuations are encountered (such as during the summer period), then the linear fit will predict the average over those fluctuations.

Overall, our model (13) is supported by theoretical considerations and has a good model fit. It is plausible that some of the variance that is not explained by our model, especially during the summer period, is stochastic, and thus cannot be explained by our type of model. A remaining issue are the three data points in October 2020, where the growth multiplier is larger than predicted by our model for three consecutive weeks, indicating that there must have been mechanisms in play that are *not* captured by our model.

### Comparison of our model to models in the literature

Our model results may be compared to values from the literature for some but not all of the models introduced in “Relationship between COVID-19 and mobility world-wide” section. For some models a comparison is not possible as they report the quality of the fit but omit information necessary to reconstruct the full model. For other models the slope is reported, but not the intercept (models by^11,12^). In these cases, we use the base R-value of our model, $R_{0, winter} = 1.4$, but point out that this limits the comparability (Table 4). Finally, for some of the models all necessary coefficients are available, thus allowing comparison to our model (models by^9,10^).

To compare the influence of mobility reduction across models, we choose the exemplary scenario in which the out-of-home duration is decreased by 40%. For the detailed comparison to each of the models^9–12^ see Supplementary Section 6.10. The model comparison yields three findings:

**Table 4:** Comparison of reduction of the effective R-value $\Delta R$ when we assume a reduction of the out-of-home duration by 40% across models and comparison of the resulting R-values when we assume an out-of-home duration of 0.

Model	^9^	^10^	^11^	^12^	Our model (12)
$\Delta R$	− 0.44	− 0.42	− 0.53	$[-0.7,-0.36]$	$[-0.75,-0.6]$
$R_t$ at ${\textbf {D}}= 0$	0.21	0.1	0.43	$-0.35$	0

^9^ provide $R_{0,i} = 0.97$, allowing us to compute $R_t$ at ${\textbf {D}}= 0$. In^10^ a plot of the regression line can be found in the supplementary material. We computed $R_t$ at ${\textbf {D}}= 0$ after reading the intercept from the plot and again under the assumption that the average at-home duration is 16 h.^11,12^ do not provide a value for the intercept. Consequently, in these cases we base the computation of the effective R-value if the out-of-home duration **D** is equal to zero (second line) on our $R_{0,winter}=1.4$ (See Supplementary Section 6.10, equation (4)).

**Figure 7:**
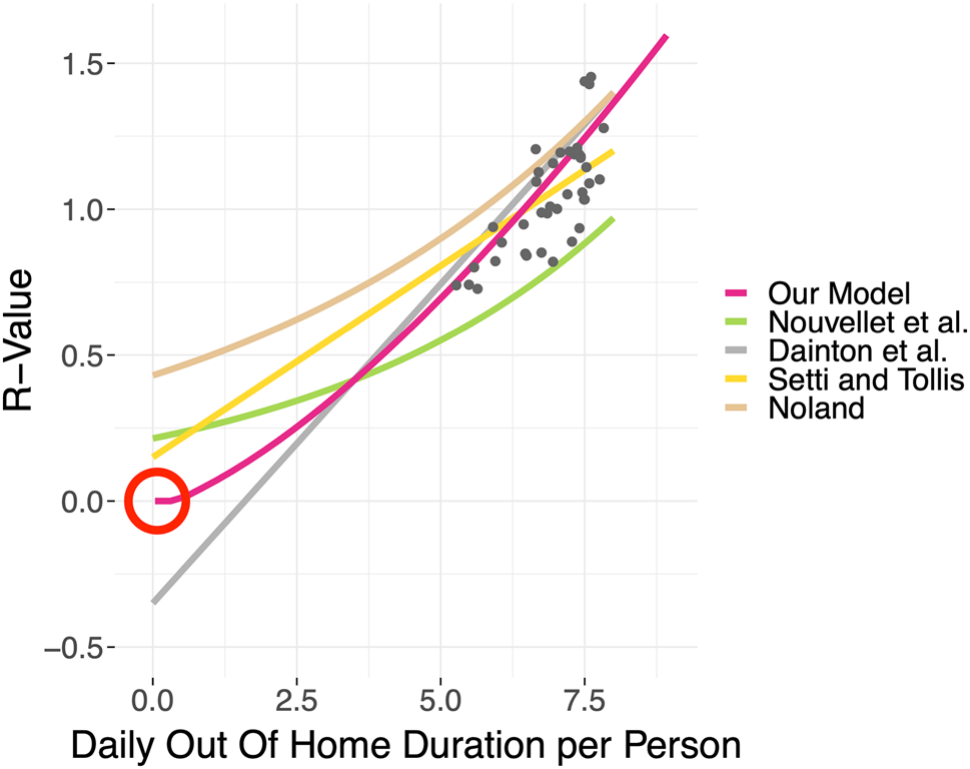
R-value vs. average daily out-of-home duration per person (in hours). Depicted are our quadratic model (12), the models by Nouvellet et al.^9^, Setti and Tollis^10^, Noland^11^, and Dainton et al.^12^. For our model, the growth multiplier was converted to the R-value, while for the models by^9–12^, the percentage change in mobility was converted to the absolute out-of-home duration (in hours). For^11,12^, as no intercept is provided, we used $R_{0,winter}=1.4$, which implies that the actual regression lines by^11,12^ are potentially shifts of our depictions. The red circle marks the origin, through which all models should plausibly go.

*First*, all models, including our own, find a reduction of the effective *R*-value between $-0.36$ and $-0.75$ if the daily out-of-home duration is reduced by 40% or 3.2 h (see Fig. 7). This, by itself, already implies a remarkable consistency across the different locations and models. Figure 7 displays the five different regression curves, and we note that to fit our data cloud, a linear slope, as used in the models by^10,12^, suffices.

*Second*, we note that when extrapolating the fit to an out-of-home duration of zero, some form of curvature is necessary to meet the requirement that the R-value is equal to zero when the mobility is zero. Without curvature, one either needs an intercept significantly below zero (grey line in Fig. 7, or the slope is not steep enough to be consistent with the data points (yellow line in Fig. 7; see also Fig. 5). – For the curved models by^9^ (green curve) and^11^ (brown curve), it is unclear if they could be adapted such that they both have the correct slope through the data *and* go through the origin.

Overall, this implies that these models are valid in the range of values where they are estimated, but do not extrapolate well beyond that range. In contrast, our quadratic model computes a growth multiplier (and thus R-value) of zero when the out-of-home duration is zero and thus allows for plausible extension to the case when the mobility is zero.

*Third and last*, we point out that the preceeding two arguments are consistent with “Mobility only models” section and especially Fig. 5, in which we already argue for a quadratic relationship between time spent outside one’s home and the growth multiplier/R-value.

In conclusion, our own model goes beyond the existing models, since we start from a hypothesis about the underlying mechanism, and from there develop a theory (see “Mobility only models” section). In consequence, our model is easier to adapt to changing circumstances.

### Consequences for disease management and extension to other communicable diseases

A quadratic reduction of the number of infections as a consequence of mobility reductions is most consistent with the data. This is also consistent with the theory developed in “Theory: relating mobility to the growth multiplier” section, which states that, if the base turnout at a gathering is reduced to a fraction $\alpha < 1$, then both the contagious and susceptible fractions are reduced, leading to a reduction of infections to $\alpha \cdot \alpha < \alpha$. One question that arises is if this is related to the disease transmission mechanism. “Theory: relating mobility to the growth multiplier” section has already pointed out that the result would be different if activity types were not reduced equally, but selectively. Similarly, the result would be different if the number of susceptible persons with which contagious persons interact would not be reduced – then the reduction factor of $\alpha$ would no longer apply to the susceptibles. For COVID-19, a possible scenario for this would be a workplace where, for a turnout of $\alpha$, also a fraction $1-\alpha$ of the available offices would no longer be used, crowding the remaining employees in only $\alpha$ of the office space. In that situation, the number of contagious persons would be reduced by $\alpha$, but the number of persons each of them encounters would be the same as before. A similar situation exists with schools: If the turnout for each class is reduced to $\alpha = 0.5$, then the number of infections is reduced to $0.5 \cdot 0.5 = 0.25$. If, however, a scheme of “alternating classes” is used (as was done in Germany, see for example^33^) , then the situation is similar to the offices above since half of the classrooms are no longer used, and in the other half of classrooms, the reduced number of contagious persons interacts with the same number of susceptible persons as without the “alternating classes” intervention.

This line of thinking extends to different transmission mechanisms: For aerosol transmission, to first order one may assume that every susceptible person in the room obtains the same dose (since breathed-out aerosols have a tendency to first rise to the ceiling and from there distribute through the room^34,35^). For droplet infection, one may assume that only persons in close distance can become infected – here, only if the number of susceptible persons within close distance is also reduced by $\alpha$, the quadratic relationship holds. As a rule of thumb, reducing person densities will lead to the quadratic reduction, whereas fully shutting down activities while maintaining others at the same person densities as before will lead to an only linear reduction – and this is expected to be largely independent from the exact mechanism of disease transmission.

## Discussion

Using a multiple linear regression model, we look into how mobility, temperature, and the COVID-19 growth multiplier are related. We find that more than 50% of the variance of the growth multiplier can be explained by the out-of-home duration alone. Furthermore, for most data sets, including our own, the elasticity of the growth multiplier with respect to changes of the out-of-home duration is larger than one. This means that a linear fit of such data points extrapolates, for out-of-home durations of zero, to negative growth multipliers – which is not plausible. In contrast, a quadratic model fits the data well, generates a growth multiplier of zero at out-of-home durations of zero, and can be motivated by theory.

Temperature alone was a worse predictor of the growth multiplier than the out-of-home duration. Temperature does provide, however, a good correction to the out-of-home duration, leading to a regression that explains more than 60% of the variance of the growth multiplier.

The growth multiplier follows changes in mobility with a delay of about 2.5 weeks. As there is only a 5-day period from infection to showing symptoms, this is longer than expected. A plausible explanation is that the German reporting system added considerable delay.

In this work, we use the growth multiplier *G* instead of the more well-known effective *R*-value as our response variable. This is motivated by the fact that for the effective *R*-value we would have to use external estimates (for example, see^36,37^), while the growth multiplier can be directly inferred from the readily available incidence data. We have computed a base growth multiplier of $G_0 = 1.54$, which translates to a base reproduction value of $R_0 \approx 1.4$ (see “Comparison of our model to models in the literature” section). In the literature, the base reproduction number $R_0$ for COVID-19 ranges between 1.5 and 3^38,39^. Although some authors argue that these estimations need to be adjusted for the increase in RT-PCR tests^40^, our value is on the lower end of what can be found in the literature. A possible reason for this is that there may be additional effects, such as people being careful, people wearing masks, etc., which may have remained in place during summer 2020 even during phases of large mobility.

In general, our proposed linear-in-parameters regression model performs well. Much of the remaining variance not explained by the model can be credited to random fluctuations due to small numbers during low incidence phase (see Fig. 6b). However, the model has systematic problems in the middle of October 2020: Fig. 6a,b show that our model underestimates the observed growth multiplier for three successive weeks in October, that is, mobility and temperature do not suffice to predict the sharp increase in case numbers. It appears that an additional mechanism, not accounted for by our model, is at play.

To this end, we explored if **school holidays** were responsible for the sharp increase. In Germany, the duration and time of school holidays depends on the federal state. In 2020, twelve out of sixteen federal states closed their schools for two weeks in fall, with Hamburg, Hesse, and Schleswig-Holstein starting on 05/Oct, Berlin, Brandenburg, Bremen, Lower Saxony, North Rhine-Westphalia, Rhineland-Palatinate, Saarland starting on 12/Oct, and Saxony and Thurungia on 19/Oct. The remaining four states only closed their schools for a single week: Mecklenburg-Vorpommern starting on 05/Oct, Saxony-Anhalt on 19/Oct and Baden-Württemberg and Bavaria starting on 26/Oct and 2/Nov respectively. Because of the time lag between exposition to the virus and the reporting of cases, manifested in our time lag of 2.5, even a school closure as early as 5/Oct is too late to explain an increase of the growth multiplier in the week of 11/Oct. Also, in Germany school holidays typically led to a *reduction* of infections – not only because schools were removed as infection contexts, but also because many parents no longer went to work during school holidays.

Another possible influence might be **disease import** when returning from vacations. However, the earliest plausible time people could return in larger numbers is after the first week of school holidays, i.e. around 12/Oct. Clearly, this is again too late to have an influence on the growth multiplier in the week of 11/Oct.

Another factor to consider is **testing**. On 03/November/2020, the testing criteria in Germany were adapted. In consequence, the number of tests of 1.602.839 in calendar week 45 dropped to 1.390.324 in calendar week 46, and the German federal health agency RKI states that the testing numbers before and after the adaptation cannot be directly compared^41^. We acknowledge that this may influence our fit for the final weeks of 2020, but because of the time discrepancy reject adapted testing policies as the reason for the described underfit during the beginning of the fall wave.

We also explored if a **sudden temperature** drop at the end of August (see Fig. 3) might have led to people moving their activities indoors despite higher temperatures at the end of September. In fact, the Google mobility data^42^ shows that the visits to parks sharply declined in mid-September and did not increase again within the study period. In consequence, we modified the outdoor fraction such that it would never *in*crease during fall 2020 – i.e. once people had decided to move a certain fraction of activities indoors, they would not be moved outdoors again until the following spring. However, this did not significantly improve the fit of our model. Overall, we do not want to rule out that indoors/outdoors behavior might have played a role here. However, neither temperature nor park usage is sufficient to explain our three sequential outliers in October 2022.

Finally, we want to identify some limitations of our model. First, we have set up and tested our model solely for Germany. Our model should be applied to more European, or even world-wide countries to explore potential differences in results between countries. Here, one should carefully explore if the translation from maximum temperature to share of activities performed outside is universal or if cultural differences have to be taken into account.

Second, the influence of weather on the disease dynamics is complex: In our model, the transformation of temperature, only used in combination with the out-of-home duration, relates to the population’s behavior and negatively correlates with the disease indicator. Other studies do not differentiate between the behavioral effect and temperature’s effect on the properties of the virus itself and/or respiratory uptake. Consequently, conflicting results regarding the first pandemic wave exist, ranging from a negative correlation between temperature and disease spread^43^, to a positive correlation^44,45^, to no evidence of a relationship between COVID-19 cases and temperature^46^. Future work should carefully disentangle the different implications of “temperature” in these works and compare them. Furthermore, as shortly noted in the beginning of the introductory paragraph of “Models including outdoor fraction” section, temperature is only one of multiple environmental mechanisms influencing the disease dynamics. We also tested for the influence of precipitation, but excluded the corresponding factors from the final model due to their statistical insignificance (see supplementary material for details). Cloudiness and humidity, which were among the strongest drivers for increased disease transmission in^47^, were not investigated in our work.

Third, as already stated in “Theory: relating mobility to the growth multiplier” section, we concentrate on the “mechanical” effect of out-of-home duration to disease spread. This leaves it open if increased out-of-home duration is due to relaxed NPIs, temporarily smaller incidences, or pandemic fatigue^7^.

Fourth, we acknowledge that an out-of-home duration (and thus a growth multiplier) of zero is a hypothetical state, which was never reached during the COVID-19 pandemic. Work by^48,49^ among others, discusses the actual minimal transmission rate. Finally, even if the mobility drops to zero once an infection is carried into a household, then the infection may still spread to the other household members before the infection chain is cut off. Consequently, when the mobility is equal to zero, our model simplifies the spreading dynamics.

## Conclusion

Under the assumption of a virus-limited regime, this work discusses whether the reduction of mobility, or, in other words, the reduction of the time spent outside one’s home, has a linear, quadratic, or even higher order effect on infection spread. If mobility is reduced by completely shutting down one activity type, but leaving the attendance of another (with the same characteristics like e.g. contact intensity, room size, indoor/outdoor activity, duration) untouched, the effect is linear. If, however, attendance of various activities is reduced evenly, a quadratic effect is observed. Finally, if mobility reductions correlate with other infection-suppressing behaviors (such as mask-wearing), even higher order effects are possible. In this work, this theory is empirically tested for the first year of the COVID-19 pandemic in Germany, demonstrating a quadratic relationship in the aforementioned country.

### Supplementary Information

Supplementary Information.

## Data Availability

All data used in this study is publicly available: RKI (incidence data): The 7-day incidence/100,000 inhabitants in Germany is provided by the Robert-Koch-Institut^24^. Meteostat (temperature data): Meteostat^26^ provides weather data on a daily basis, which served as the basis for **Tmax** (and consequently **outFrac**), **Tavg** and **Precip**. Senozon and TU Berlin (mobility data): The weekly out of home duration is available on Zenodo^25^. Google (alternative mobility data source): Usage of Google COVID-19 Community Mobility reports in the supplementary material^42^. Google stopped reporting new data on October 15, 2022, but all published data is still publicly available.

## Code availability

All code used to produce the results is publicly available on Zenodo^50^.
