# Supplementary material for: The effect of mobility reductions on infection growth is quadratic in many cases
Source: Sci Rep. 2024 Jun 24;14:14475. doi: 10.1038/s41598-024-64230-1 (PMC11196635; doi:10.1038/s41598-024-64230-1)
Supplement: Supplementary file 1 — Supplementary Information. [file 41598_2024_64230_MOESM1_ESM.pdf]

The effect of mobility reductions on infection growth is quadratic in many cases - Supplementary Material

Sydney Paltra[1,*], Inan Bostanci[2], and Kai Nagel[1]

[1]Technische Universität Berlin, FG Verkehrssystemplanung und Verkehrstelematik, 10623 Berlin, Germany
[2]Zuse Institute Berlin, 14195 Berlin, Germany
[*]correspondence: paltra@tu-berlin.de

1 Supplementary material

1.1 Simulation Study - Quadratic Effect of Mobility Reductions

To further substantiate our claim that the effect of mobility reductions is *quadratic* in many cases, we implemented a toy model. The agent-based toy model was written in Julia v1.9.2 [1], heavily relies on Agents.jl [2], a framework for agent-based models, and is freely available on GitHub `https://github.com/matsim-vsp/infoXpand/tree/main/Task1/Julia`.

In our toy model, we reconsider the example introduced in Section "Theory: relating mobility to the growth multiplier": Assume, there are 400 agents who all live in single-person households and whose only non-home activity is work in an open-plane office. Every day (in other words: every time step) the agents go to work for a long period of time. If among the agents is an contagious agent, they emit virus particles and thus risk infecting their coworkers. These virus particles are uniformly mixed into the air volume, and consequently, a contagious agent may infect a susceptible agents with infection probability π.

In the simulation runs, turnout α ($\in [0.1, 1]$) and infection probability π ($\in \{0.01, 0.02, 0.03, 0.04, 0.05\}$) are varied. For each combination of turnout and infection probability we run 100 simulations; in each simulation, patient zero, who is infected at the beginning of the simulation, is newly chosen. Furthermore, as the infectious agents also follows the reduced office attendance, α also denotes probability with which patient zero will be at the office. The expected no. of infected individuals for different values of α is depicted in Figure 1:

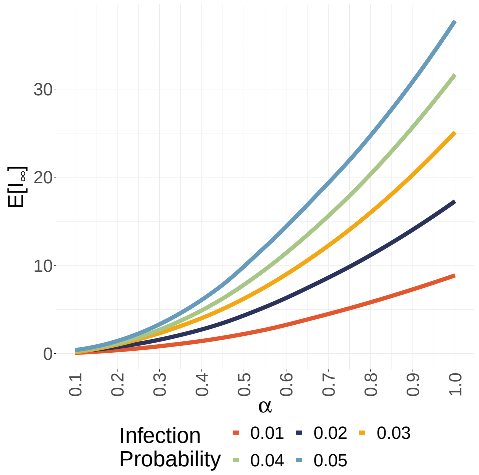

Figure 1: Expected no. of infected individuals vs. share of attendees α after 2000 time steps.

1.2 Relationship of COVID-19 and Mobility in Germany

As noted in Section "Relationship between COVID-19 and mobility in Germany", multiple studies investigate the reduction of mobility in Germany related to the COVID-19 pandemic:

Conducting an analysis of movement records from mobile phones, [3] found that lockdown measures caused substantial and long-lasting structural changes in mobility, noting a heterogeneous spatial distribution of reductions (increased reduction in large cities compared to less densely populated areas) and the largest reduction for mid-March 2020. By analyzing the Google mobility data [4] for the federal state of Saxony until July 2020, [5] found an overall decline in mobility for all commercial activities and an increase for parks and residential locations. Additionally, using the three mobility categories (transit, walking driving) of the Apple mobility data [6], [7] found a negative correlation between mobility and confirmed case numbers in Germany.

Using survey data from the first wave of the COVID-19 pandemic in Germany, [8] found the most significant mobility reduction for recreational trips followed by work trips. Here, public transit displays the largest decrease in share, in terms of modal split. [9] carried out a self-assessment survey, noting that the share of the adult population which had public transport in their mode choice set had dropped by almost half during the lockdown of spring 2020. Finally, concentrating on the rural population and using data from interviews and a survey, [10] found that a considerable share of respondents stated to not have changed their mobility behavior, and that in consequence insights from urban settings cannot be directly transferred to rural areas.

Returning to the question of mode choice, [9] investigated the car usage in Germany before (spring 2019) and during (spring 2020) the pandemic, finding an effect as overall mileage decreased (magnitude of decrease depended on household characteristic, age and cubic capacity of the car), but noting that at the time of writing it was unclear whether or not these changes will turn out to be structural.

Furthermore, [11] computed the change in general mobility behavior at the Germany county level in January 2021 compared to the same period of the previous year, finding that mobility changes show significant socioeconomic heterogeneity. Contrarily, analyzing continuous location (provided via a smartphone app, from beginning of March until mid-May 2020) in combination with demographic characteristics, [12] found that the German population reduced its mobility in a rather consistent and uniform way. They found no evidence for statistically significant period-by-subgroup interactions. In a follow-up research letter, [13] additionally analyzed the reduction in daily distance travelled by an individual person up until June 2021, again finding that the pattern of relative reduction was similar in all age groups and in both sexes, with a slightly higher relative mobility in the younger age group in summer 2020 and the last month of observation.

1.3 Comparison of Mobility Data Sources

In this work, we have used the out-of-home duration by Senozon [14]. Alternative data sources are the Google COVID-19 Community Mobility Reports. The google mobility reports provide mobility data for six different categories, including "residential". Mobility data is presented relative to a baseline value. For each day of the week, the baseline value is the median value from the 5-week period from January 3rd until February 6th, 2020. Assuming that an average person in Germany spends around 15.7 hours per day at home [15], the relative numbers for the category "residential" can be converted to absolute numbers, i.e. the amount of time people spent at home. From the absolute amount of time spent at home, one can then infer the amount of time people did not spent at home. This is then comparable to the number we have used above. Please note that when trying to apply our method to other nations, another assumption for the baseline out-of-home duration might be necessary.

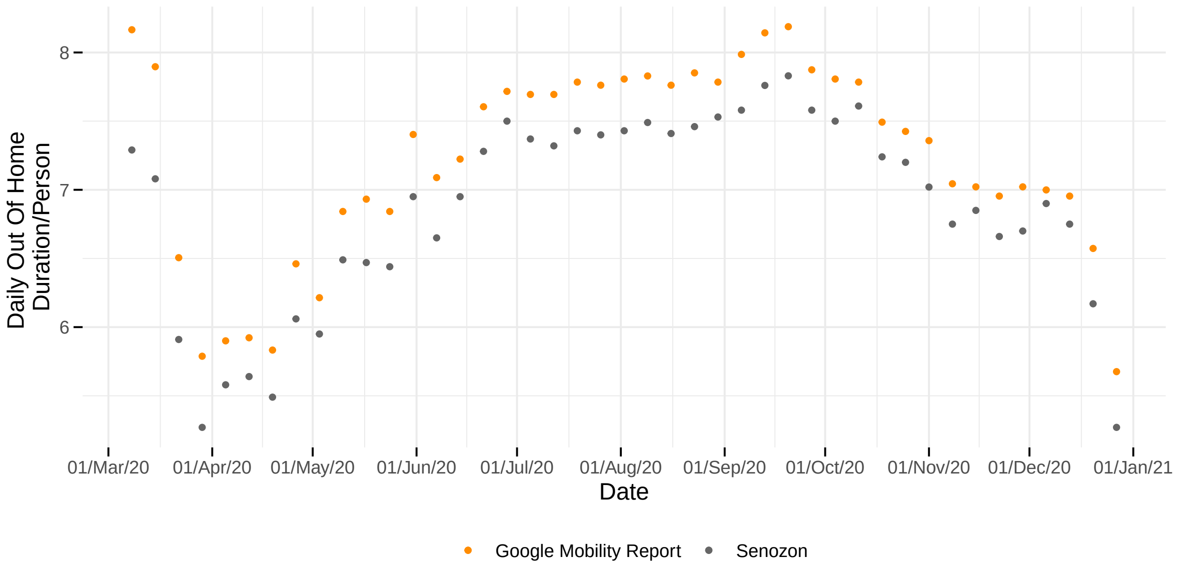

Figure 2: Daily out of home duration per person (in hours) for 2020. Comparison of Senozon and Google COVID-19 Community Mobility Reports data.

1.4 Model Comparison (Schematic Description)

To settle on the final model (12), we employ a multi-step model comparison (see also Figure 3):

- In the first step, we consider mobility-only models (see Section "Mobility only models" for details), to determine if the influence of mobility on the growth multiplier was linear, quadratic or cubic.
- Then, we compare the intercept and no-intercept model arguing for the exclusion of the intercept (again, see Section "Mobility only models").
- In the third step, we compare multiple weather variables: the weekly average of the daily maximum temperature **Tmax**, the weekly average of the daily average temperature **Tavg**, the weekly average of the daily total precipitation **Precip**, and the outdoor fraction **outFrac**. Model outputs and detailed discussion of this step can be found in the Supplementary Section 1.5.
- In the final step, we exclude the linear term for **outFrac** and only include the interaction term of out-of-home duration and **outFrac** (see Section "Models including outdoor fraction").

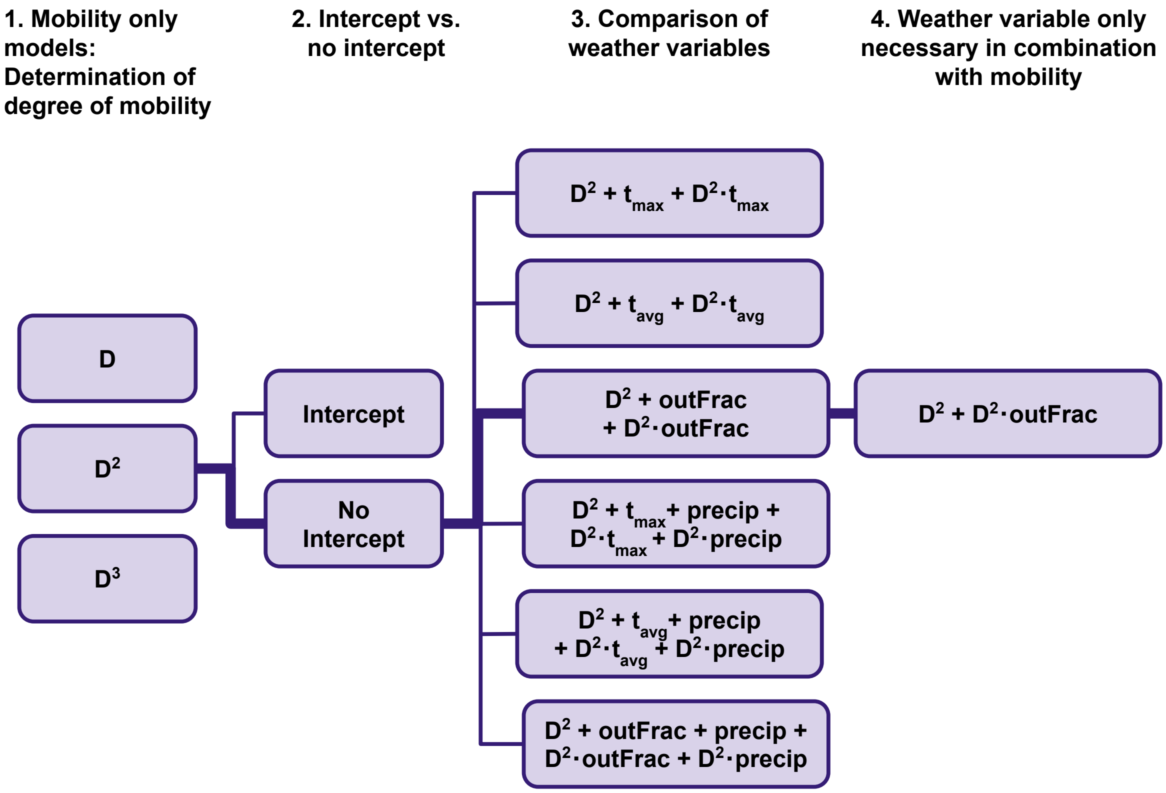

Figure 3: Multi-step model comparison to determine the final model (12).

1.5 Model Comparison (Model Outputs)

Step 1 - Mobility only models

In a first step, we compare multiple mobility only models:

$$\mathbf{G}_{t+2} = \beta_0 + \beta_d \mathbf{D}_t^m.$$

Here, the growth multiplier is denoted by $\mathbf{G}_t = I_{t+1}/I_t$, where I_t is the weekly average incidence, t indexes the weeks, the out-of-home duration is denoted by $\mathbf{D}_t$, and for which $m \in \{1, 2, 3\}$. Based on the arguments detailed in Section "Mobility only models", we decide on the quadratic model ($m = 2$). For details and model outputs, see Section "Mobility only models".

Step 2 - Intercept vs. no Intercept

In the second step, we compare the quadratic intercept and no-intercept model:

$$\mathbf{G}_{t+2} = \beta_0 + \beta_d \mathbf{D}_t^2,$$
$$\mathbf{G}_{t+2} = \beta_d \mathbf{D}_t^2, \tag{1}$$

Again, the model outputs and our reasoning for continuing with the no-intercept model (1) are provided in Section "Mobility only models".

Step 3 - Comparison of weather variables

As noted in Section "Models including outdoor fraction", the inclusion of the share of activities performed outside (**outFrac**) instead of temperature itself, is based on our previous modelling work. To additionally support this variable transformation, we set up analogous models to equation (11), but

with the weekly average of the daily maximum temperature **Tmax**, or the weekly average of the daily average temperature **Tavg** instead of **outFrac**. For **Tmax**, this model reads

$$\mathbf{G}_{t+2} = \beta_{d^2}\mathbf{D}_t^2 + \beta_{temp}\mathbf{Tmax}_t + \beta_{tempd^2}\mathbf{D}_t^2 \cdot \mathbf{Tmax}_t$$

and creates the output found in the following table:

Variable	Coefficient	Std. Error	$p > \|t\|$	[95% CI]
$\mathbf{D}^2$	0.0268203	0.0019012	$< 2e-16$	$(0.0230, 0.0307)$
**Tmax**	-0.0067756	0.0095029	0.480	$(-0.0260, 0.0125)$
$\mathbf{D}^2 \cdot \mathbf{Tmax}$	-0.0001027	0.0001719	0.554	$(-0.0005, 0.0002)$
Adjusted R^2	0.5956			
AIC	-21.2415			
BIC	-14.48598			

Table 1: Model output, out-of-home duration **D** and temperature **Tmax** lead by 2 weeks.

Analogously, for **Tavg** the corresponding model reads

$$\mathbf{G}_{t+2} = \beta_{d^2}\mathbf{D}_t^2 + \beta_{temp}\mathbf{Tavg}_t + \beta_{tempd^2}\mathbf{D}_t^2 \cdot \mathbf{Tavg}_t,$$

which leads to the output found in Table 2.

Variable	Coefficient	Std. Error	$p > \|t\|$	[95% CI]
$\mathbf{D}^2$	$2.652e-02$	$1.738e-03$	$< 2e-16$	(0.0230, 0.0300)
**Tavg**	$-1.367e-02$	$1.471e-02$	0.359	$(-0.0435, 0.0161)$
$\mathbf{D}^2 \cdot \mathbf{Tavg}$	$-3.123e-05$	$251e-04$	0.903	$(-0.0005, 0.0005)$
Adjusted R^2	0.5992			
AIC	-21.59887			
BIC	-14.84335			

Table 2: Model output, out-of-home duration **D** and temperature **Tavg** lead by 2 weeks.

For comparison, the model for the outdoor fraction **outFrac**, which we presented in Section "Models including outdoor fraction", reads

$$\mathbf{G}_{t+2} = \beta_{d^2}\mathbf{D}_t^2 + \beta_o\mathbf{outFrac}_t + \beta_{od^2}\mathbf{D}_t^2 \cdot \mathbf{outFrac}_t. \quad (2)$$

and its result can be found in Table 3.

Variable	Coefficient	Std. Error	$p > \|t\|$	[95% CI]
$\mathbf{D}^2$	0.0242	0.0008	$< 2e-16$	$(0.0226, 0.0259)$
**outFrac**	0.0167	0.3976	0.967	$(-0.7889, 0.8222)$
$\mathbf{D}^2 \cdot \mathbf{outFrac}$	-0.0053	0.0074	0.478	$(-0.0204, 0.0097)$
Adjusted R^2	0.6345			
AIC	-25.2865			
BIC	-18.5310			

Table 3: Model output of (2), out-of-home duration **D** and outdoor fraction **outFrac** lead by 2 weeks.

Besides temperature (and its transformation **outFrac**), it is possible that precipitation influences behavior and therefore, indirectly, the growth multiplier **G**. We consider a model that includes the variable precipitation (**Precip**) and its interaction with the squared out-of-home duration $\mathbf{D}^2$. For **Tmax**, this model reads

$$\mathbf{G}_{t+2} = \beta_{d^2}\mathbf{D}_t^2 + \beta_{temp}\mathbf{Tmax}_t + \beta_p\mathbf{Precip} + \beta_{tempd^2}\mathbf{D}_t^2 \cdot \mathbf{Tmax}_t + \beta_{pd^2}\mathbf{D}_t^2 \cdot \mathbf{Precip}_t$$

and produces the model output found in Table 4.

Variable	Coefficient	Std. Error	$p > \|t\|$	[95% CI]
$\mathbf{D}^2$	0.0266581	0.0020713	$7.84e-15$	$(0.0224532100, 0.0308630041)$
**Tmax**	0.0009636	0.0116898	0.935	$(-0.0227679034, 0.0246950782)$
**Precip**	-0.1612546	0.1498053	0.289	$(-0.4653755335, 0.1428662861)$
$\mathbf{D}^2 \cdot$ **Tmax**	-0.0002581	0.0002195	0.248	$(-0.0007037921, 0.0001874938)$
$\mathbf{D}^2 \cdot$ **Precip**	0.0032761	0.0028741	0.262	$(-0.0025586248, 0.0091107965)$
Adjusted R^2	0.6111			
AIC	-18.80341			
BIC	-8.670129			

Table 4: Model output, out-of-home duration **D**, temperature **Tmax**, and precipitation **Precip** lead by 2 weeks.

For **Tavg**, the corresponding model reads

$$\mathbf{G}_{t+2} = \beta_{d^2}\mathbf{D}_t^2 + \beta_{temp}\mathbf{Tavg}_t + \beta_p\mathbf{Precip} + \beta_{tempd^2}\mathbf{D}_t^2 \cdot \mathbf{Tavg}_t + \beta_{pd^2}\mathbf{D}_t^2 \cdot \mathbf{Precip}_t$$

with the output found in Table 5.

Variable	Coefficient	Std. Error	$p > \|t\|$	[95% CI]
$\mathbf{D}^2$	0.0262721	0.0018416	$3.75e-16$	$(0.022533560, 0.0300106975)$
**Tavg**	-0.0014775	0.0181616	0.936	$(-0.038347507, 0.0353925863)$
**Precip**	-0.1631482	0.1524124	0.292	$(-0.472561924, 0.1462654806)$
$\mathbf{D}^2 \cdot$ **Tavg**	-0.0002820	0.0003317	0.401	$(-0.000955435, 0.0003914227)$
$\mathbf{D}^2 \cdot$ **Precip**	0.0034440	0.0029206	0.246	$(-0.002485039, 0.0093730891)$
Adjusted R^2	0.6184			
AIC	-19.5587			
BIC	-9.425426			

Table 5: Model output, out-of-home duration **D**, temperature **Tavg**, and precipitation **Precip** lead by 2 weeks.

Finally, for the outdoor fraction **outFrac**, the model reads

$$\mathbf{G}_{t+2} = \beta_{d^2}\mathbf{D}_t^2 + \beta_o\mathbf{outFrac}_t + \beta_p\mathbf{Precip} + \beta_{od^2}\mathbf{D}_t^2 \cdot \mathbf{outFrac}_t + \beta_{pd^2}\mathbf{D}_t^2 \cdot \mathbf{Precip}_t \tag{3}$$

and its output can be found in Table 6.

Variable	Coefficient	Std. Error	$p > \|t\|$	[95% CI]
$\mathbf{D}^2$	0.024256	0.001133	$< 2e - 16$	(0.021955690, 0.026555956)
**outFrac**	0.201940	0.426215	0.639	$(-0.663322464, 1.067201797)$
**Precip**	-0.149454	0.122266	0.230	$(-0.397667222, 0.098759067)$
$\mathbf{D}^2 \cdot \mathbf{outFrac}$	-0.009031	0.008045	0.269	$(-0.025363158, 0.007300653)$
$\mathbf{D}^2 \cdot \mathbf{Precip}$	0.002909	0.002319	0.218	$(-0.001799981, 0.007616995)$
Adjusted R^2	0.6502			
AIC	-23.05226			
BIC	-12.91899			

Table 6: Model output, out-of-home duration **D**, outdoor fraction **outFrac**, and precipitation **Precip** lead by 2 weeks.

We note that model (3) has the highest adjusted R^2 (0.6502), directly followed by model (2) (0.6345). However, for model (2), both AIC and BIC display lower values. Consequently, we decide to continue with model (2).

Step 4 - Only considering weather in combination with mobility

In Section "Models including outdoor fraction", we highlighted statistically and theoretically motivated reasons for excluding the linear term **outFrac** (see Section "Models including outdoor fraction" for details). The model from the aforementioned section was

$$\mathbf{G}_{t+2} = \beta_{d^2}\mathbf{D}_t^2 + \beta_{od^2}\mathbf{D}_t^2 \cdot \mathbf{outFrac}_t$$

and had the output shown in Table 7.

Variable	Coefficient	Std. Error	$p > \|t\|$	[95% CI]
$\mathbf{D}^2$	0.0242	0.0008	$< 2e - 16$	(0.0226, 0.0258)
$\mathbf{D}^2 \cdot \mathbf{outFrac}$	-0.0050	0.0015	0.0017	$(-0.0081, -0.0020)$
Adjusted R^2	0.6345			
AIC	-27.2846			
BIC	-22.2180			

Table 7: Model output for final model, out-of-home duration **D** and outdoor fraction **outFrac** lead by 2 weeks.

1.6 Model Comparison (Multiple Mobility Terms Simultaneously)

In addition to considering the linear, quadratic, and cubic out-of-home duration separately, we also considered them simultaneously using orthogonal polynomial regression. The model output can be found in Table 8.

Variable	Coefficient	Std. Error	$p > \|t\|$	[95% CI]
Intercept	1.07306	0.03023	$< 2e-16$	(1.0118, 1.1344)
$\mathbf{D}$	1.23506	0.19119	$1.7e-07$	(0.8473, 1.6228)
$\mathbf{D}^2$	0.12990	0.19119	0.501	$(-0.2579, 0.5177)$
$\mathbf{D}^3$	0.06433	0.19119	0.738	$(-0.3234, 0.4521)$
Adjusted R^2	0.5019			
AIC	-13.0579			
BIC	-4.6135			

Table 8: Model output for model including linear, quadratic, and cubic out-of-home duration. The out-of-home duration leads by 2 weeks.

We note that considering the out-of-home duration up to degree three does not improve the model fit (see Table 2 for regression output for linear, quadratic, and cubic mobility-only model). Consequently, we endorse the models including only a single mobility term. Furthermore, we want to point out that the model considering the linear, quadratic, and cubic term simultaneously is also considered and dismissed in the best subset selection in Section 1.8.1.

1.7 Model Comparison (Determination of Lag)

To determine that the lag between growth multiplier and out-of-home-duration is 2 weeks, we considered every model of every step of Figure 3 with a lag of 0 to 4 weeks. When considering the mobility-only models (with or without intercept), a lag of three weeks leads to the best model fit. However, after the inclusion of a weather variable, a 2-week lead is favored. Summary statistics can be found in the following Table 9:

Step	Model	Lag	Adj. R^2	AIC	BIC
1	Linear	0	0.0054	85.8698	90.9365
		1	0.2664	10.9137	15.9803
		2	0.5207	−16.4284	−11.3618
		3	0.5526	−19.6422	−14.5756
		4	0.4857	−13.2082	−9.3147
1	Quadratic	0	0.0044	85.9096	90.9763
		1	0.2645	11.0201	16.0868
		2	0.5374	−16.8026	−11.7359
		3	0.5791	−21.0451	−15.9783
		4	0.5117	−15.4195	−10.3529
1	Cubic	0	0.003	85.9634	91.0301
		1	0.2605	11.2376	16.3043
		2	0.527	−16.9506	−11.8839
		3	0.5807	−22.2352	−17.1685
		4	0.5105	−16.3578	−11.2911
2	No-Intercept Quadratic	0	0.0053	84.9116	88.2893
		1	0.2458	10.0256	13.4034
		2	0.5250	−18.7982	−15.4204
		3	0.5675	−22.9995	−19.6218
		4	0.4980	−17.3541	−13.9764
$3^\star$	$\mathbf{D}^2 + \mathbf{outFrac} + \mathbf{D}^2 \cdot \mathbf{outFrac}$, no intercept	0	0.0840	84.5769	91.3324
		1	0.4650	0.2894	7.0449
		2	0.6344	−25.2658	−18.5103
		3	0.5769	−19.8711	−13.1155
		4	0.5025	−13.7148	−6.9593
4	$\mathbf{D}^2 + \mathbf{D}^2 \cdot \mathbf{outFrac}$, no intercept	0	0.0820	82.6628	87.7295
		1	0.4637	−1.6182	3.4484
		2	0.6344	−27.2639	−22.1972
		3	0.5758	−21.7689	−16.0722
		4	0.4980	−15.3549	−10.2883

Table 9: Regression output for different lags for the different steps of the model comparison illustrated in Figure 3. $3^\star$: All models of step 3 of Figure 3 were compared for a lag of 0-4 weeks. However, for all lags the model including the outdoor fraction **outFrac** was favored and for reasons of clarity, only the summary statistics of the best-fitting model are given in the table.

1.8 Model selection via Best subset selection, Lasso regularization, elastic net regularization

In Sections "Mobility only models" and "Models including outdoor fraction" (and in Supplementary material 1.5), theoretical assumptions as well as visual results (see Figure 5) guided the model selection. Complementary, in this subsection we show that using three different variable selection methods, namely best subset selection, Lasso regularization, and elastic net regularization, supports our conclusion.

Due to the arguments presented in Sections "Mobility only models" and "Models including outdoor fraction", we consider the mobility variable **D** up the degree three, the outdoor fraction **outFrac** up to degree three and every combination of them up until degree 3, leaving us with 9 predictors ($\mathbf{D}, \mathbf{D}^2, \mathbf{D}^3, \mathbf{outFrac}, \mathbf{outFrac}^2, \mathbf{outFrac}^3, \mathbf{D}\times\mathbf{outFrac}, \mathbf{D}^2\times\mathbf{outFrac}, \mathbf{D}\times\mathbf{outFrac}^2$), and 2^9 models to choose from. Furthermore, due to the reasoning presented in Section "Mobility only models", we enforce that the intercept is equal to 0.

1.8.1 Best subset selection

Best subset selection aims to find a small subset of predictors for a linear model. When performing best subset selection, a separate least squares regression model must be fit for each possible combination the predictor variables. For every possible subset size of variables, the best subset selection chooses the model with the minimal residual sum of squares (RSS). A single best model can then chosen by using one of the following three statistics: Bayesian Information Criterion (BIC), Mallow's C_p or adjusted R^2. Here, we consider all three of them, to choose a model which best satisfies our needs. In addition, we enforce that the maximum number of independent variables is **3**.

BIC: Comparing the 2^9 possible models, the model with the smallest BIC (see Figure 4) is

$$\mathbf{G}_{t+2} = \beta_{d^2}\mathbf{D}^2 + \beta_{d^2o}\mathbf{D}^2 \cdot \mathbf{outFrac}.$$

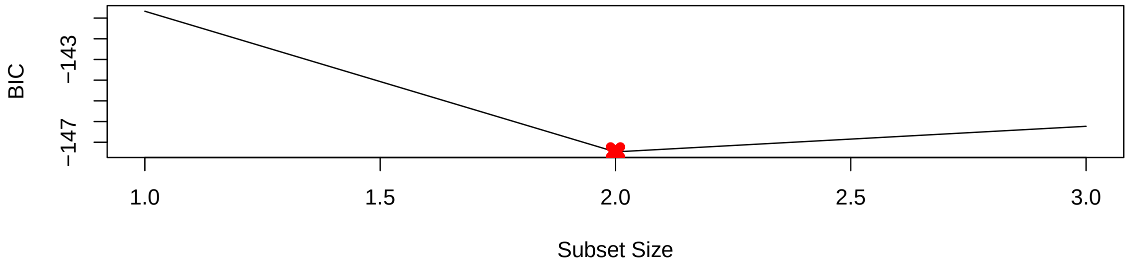

Figure 4: BIC for the different subset sizes.

Mallow's C_p: Among all models, the model with the smallest C_p (see Figure 5 is the following one:

$$\mathbf{G}_{t+2} = \beta_{d^3}\mathbf{D}^3 + \beta_{do}\mathbf{D} \cdot \mathbf{outFrac} + \beta_{d^2o}\mathbf{D}^2 \cdot \mathbf{outFrac}$$

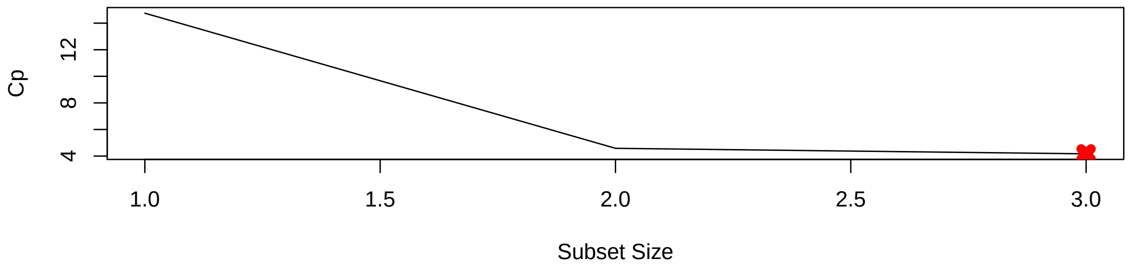

Figure 5: Mallow's C_p for the different subset sizes.

Adjusted R^2: And finally, among all 2^9, the model with the largest adjusted R^2 (see Figure 6 is as follows:

$$\mathbf{G}_{t+2} = \beta_{d^3}\mathbf{D}^3 + \beta_{do}\mathbf{D}\cdot\mathbf{outFrac} + \beta_{d^2o}\mathbf{D}^2\cdot\mathbf{outFrac}$$

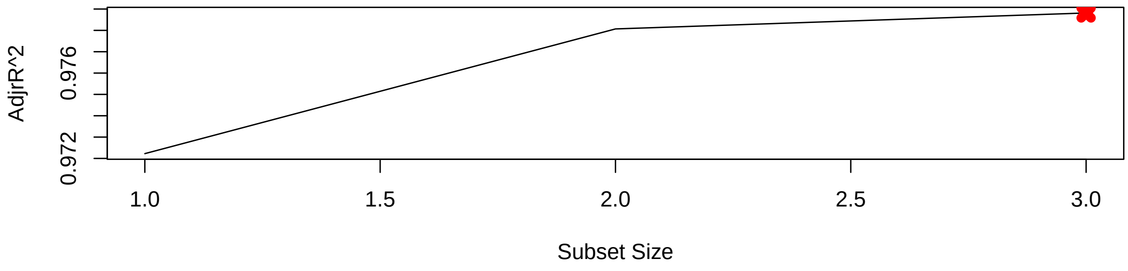

Figure 6: Adjusted R^2 for the different subset sizes. The maximal adjusted R^2 is equal to 0.6562 and reached, when the subset size is equal to 3. However, already when the subset size is equal to 2, we obtain an adjusted R^2 of 0.6345.

1.8.2 Lasso regularization

Lasso is a regression method which performs both variable selection and regularization with the aim of improving interpretability and model accuracy. In our case, Lasso regularization favors the model

$$\mathbf{G}_{t+2} = \beta_d\mathbf{D} + \beta_{d^2}\mathbf{D}^2 + \beta_{d^3}\mathbf{D}^3 + \beta_{o^3}\mathbf{outFrac}^3 + \beta_{d^2o}\mathbf{D}^2\cdot\mathbf{outFrac}$$

1.8.3 Elastic net regularization

Elastic net regularization is a penalized linear regression model combining both L_1 and L_2 penalties. In consequence, elastic net regularization can be viewed as a combination of Ridge and Lasso regression. As the degree of mixing α we chose $\alpha = 0.5$, and we chose the shrinkage parameter using 10-fold cross validation. The cross validation was performed 100 times, giving us a distribution of λs. Both mean(λ) and median(λ) favor the model

$$\mathbf{G}_{t+2} = \beta_d\mathbf{D} + \beta_{d^2}\mathbf{D}^2 + \beta_{d^3}\mathbf{D}^3 + \beta_{o^3}\mathbf{outFrac}^3 + \beta_{d^2o}\mathbf{D}^2\cdot\mathbf{outFrac}$$

1.8.4 Conclusion

Summarizing the results of the best subset selection, the lasso regularization and the elastic net regularization above, we observe that the different variable selection methods favor different models. However,

we note that all three methods (as well as the 3 different criteria when applying best subset selection) select $\mathbf{D}^2 \cdot \mathbf{outFrac}$ as an independent variable and lasso regularization, elastic net regularization as well as BIC (when applying best subset selection) also include $\mathbf{D}^2$. Contrarily, Mallow's Cp and the adjusted R^2 favor $\mathbf{D}^3$ as the mobility-only variable. Furthermore, when deciding based on BIC in best subset selection, the model $\mathbf{G}_{t+2} = \beta_{d^3} D^2 + \beta_{d^2 o} \mathbf{D}^2 \cdot \mathbf{outFrac}$ is chosen, which is the model we have already favored in Section "Models including outdoor fraction". By Occam's razor, and as best subset selection is the preferable method in this case, we decide on $\mathbf{G}_{t+2} = \beta_{d^2} \mathbf{D}^2 + \beta_{d^2 o} \mathbf{D}^2 \cdot \mathbf{outFrac}$ as the final model.

1.9 Model diagnostics

In order to assess the adequacy of the final Model (12), several model diagnostics were conducted. Figure 7 presents a scatter plot of the residuals against the predicted values from the model (top) as well as a scatter plot of the square root of the absolute value of the standardized residuals against the predicted values from the model (bottom). According to the assumption of homoscedasticity, we expect both panels to exhibit a random scatter of points around zero. However, the rightmost portion of the top panel suggests the presence of heteroscedasticity and a potential non-linear relationship. To further investigate this, a Breusch-Pagan test was performed, which examines the presence of heteroscedasticity in the residuals. The test yields a p-value of 0.1479 (> 0.05), indicating a lack of evidence for heteroscedasticity.

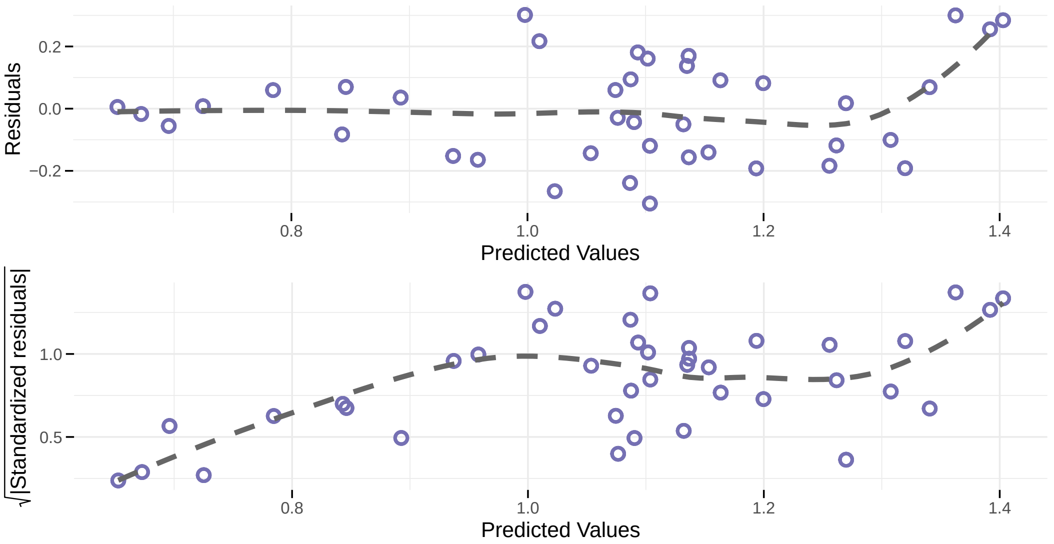

Figure 7: Top: Residuals vs. predicted values, bottom: $\sqrt{|\text{Standardized residuals}|}$ vs. predicted values.

Figure 8 (top) shows the model residual quantiles vs. the theoretical quantiles if the residuals were to follow a normal distribution. A slight deviation can be noticed at the extreme ends, while the vast majority of the model quantiles aligns well with the theoretical quantiles. Finally, we use Cook's distance to detect observations that strongly influence fitted values of the model, which can be seen in Figure 8. Observations with a Cook's distance larger than the threshold of $4/n$, where n is the number of observations, are traditionally deemed outliers. With $n = 40$, three observations pass this threshold: Observation number 33 (corresponding to 18-October-2020) displays the largest Cook's distance ($1.436696e-01$), observation 34 (corresponding to 25-October-2020) displays the second largest Cook's distance ($1.381654e-01$), and observation 32 (corresponding to 11-October-2020) displays the third-largest cook's distance ($1.092919e-01$).

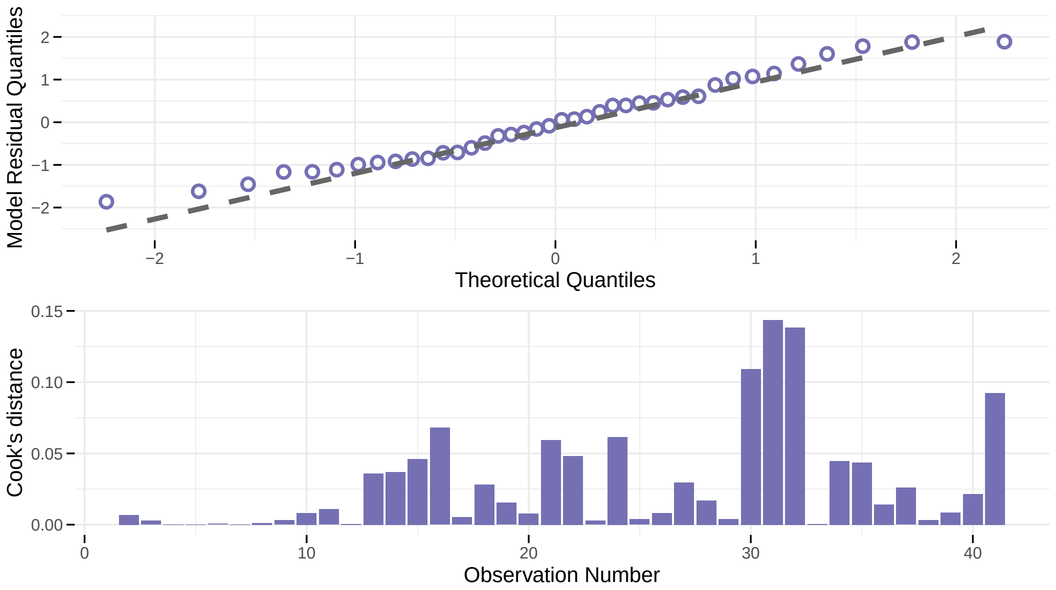

Figure 8: Top: Normal Q-Q plot, bottom: Bar plot of Cook's distance.

Altogether, the model diagnostics as well as Figure 3a and Figure 3b indicate that the model generally seems appropriate for the relationship that we aim to model, but three outliers at the beginning of the second wave are not fully explained by the proposed model, which were discussed elaborately in the Results section.

1.10 Comparison of Our Model to Models in the Literature

As address in Section "Comparison of our model to models in the literature", we compare our model values to values in the literature whenever possible. This is not possible for the publications that report the quality of the fit but omit information necessary to reconstruct the full model (see Section "Relationship between COVID-19 and mobility world-wide"). However, the following four papers provide the necessary coefficients and can thus be compared to our model.

To compare the influence of mobility reduction across models, we choose the following scenario: For all comparisons, we assume that the out-of-home duration is decreased by 40%. The average German out-of-home duration is around 8 hours per day (see Supplementary Section 1.3), leading to an average *at-home* duration of about 16 hours per day. Reducing the out-of-home duration by -40%, or $-3.2\,hrs$, to $4.8\,hrs$ thus corresponds to increasing the *at*-home-duration by 20%.

According to (13), our base growth multiplier $\mathbf{G}_{0,winter}$, at $\mathbf{D} = 8$ *hours* and $\mathbf{outFrac} = 0$, is 1.54. In order to translate the weekly growth multiplier to the R-value, the generation time needs to be taken into account. Assuming the generation time of SARS-CoV-2 as 5 days [16–18], we obtain

$$R_{0,winter} = \mathbf{D}_{0,winter}{}^{5/7} = 1.54^{5/7} \approx 1.4\ . \tag{4}$$

Assuming the aforementioned reduced out-of-home time of $8\,hrs - 3.2\,hrs = 4.8\,hrs$, we obtain

$$R_{winter}(-3.2hrs) = (\mathbf{D}_{winter}(-3.2hrs))^{5/7} = (0.024 \cdot 4.8^2)^{5/7} \approx 0.55^{5/7} \approx 0.65\ . \tag{5}$$

The corresponding reduction of the effective R-value thus is

$$\boxed{\Delta R_{winter} := R_{winter}(-3.2hrs) - R_{0,winter} \approx 0.65 - 1.4 = -0.75\ .} \tag{6}$$

The same computation, with $\mathbf{outFrac} = 1$, i.e. "during summer", leads to $\Delta R_{summer} \approx -0.6$.

Some of the models we compare with are linear, and for these models ΔR can be obtained from the slope coefficient without having to determine the intercept. In the following, we thus compare with our ΔR, which is between 0.6 (warm summer) and 0.75 (cold winter).

Comparison with [19] [19] use

$$log(R_{t,i}) = log(R_{0,i}) - \beta_i(1 - m_{t,i}), \tag{7}$$

where $m_{t,i}$ denotes the remaining fraction of mobility compared to a baseline, expressed as a value between 0 and 1. The remaining mobility fraction $m_{t,i}$ is based on various categories from Google [4] or Apple [6] mobility data. The percentage changes must then be divided by 100 to obtain $m_{t,i}$. When using a combination of google and apple mobility data, the authors obtain $\beta_i = 1.51$ and $R_{0,i} = 0.97$ for Germany during the period from late April to late October 2020. Using our introduced example, namely a reduction of the out-of-home duration of 40% (= 0.4), we obtain

$$\begin{aligned} &log(R_{Ger}) = log(0.97) - 1.51 \cdot 0.4 \approx -0.634459 \\ &\Leftrightarrow R_{Ger} \approx 0.53 \\ &\Leftrightarrow \Delta R_{Ger} \approx 0.53 - 0.97 = -0.44 \ . \end{aligned}$$

This is somewhat smaller, but still consistent with our own range of $[-0.75, -0.6]$.

Comparison with [20] [20] analyze the correlation between effective reproduction number and mobility. For the "residential" category, this leads to

$$R_t = \beta_0 + \beta_r \cdot h_{t-\tau}, \tag{8}$$

where R_t denotes the effective reproduction number and h_t denotes the percentage change of time spent at home as introduced above. For a 14-day lag ($\tau = 14$) they compute a coefficient of $\beta_r = -0.021$ for Germany. Hence, for a 20% increase of the at-home duration, their model computes a reduction of the effective R-value by $-0.021 \cdot 20 = -0.42$. Again, this is somewhat smaller, but still consistent with our own range of $[-0.75, -0.6]$.

Comparison with [21] [21] uses a fixed-effects model that controls for state-level effects to explore the relationship between Google mobility data and R_t on US state level. For the "residential" category, the model reads

$$log(R_{t,state}) = \beta_{0,state} + \beta_r \cdot h_{t-\tau} + \beta_d \cdot days_t \ , \tag{9}$$

where $\beta_{0,state}$ denotes a state-specific intercept, h_t denotes the percentage change of the "residential" category, and $days_t$ is a trend variable denoting the days since the start of the pandemic in the state. He obtains $\beta_r = -0.0236$ and $\beta_d = -0.0020$ when considering a 14-day lag ($\tau = 14$) between mobility and the effective R-value. The values of $\beta_{0,state}$ are not given in the paper; to compare with our example one can set $h_{t-\tau}$ and $days_t$ to zero and then use $\beta_{0,state} = \log R_0 = \log(1.4)$. For our example of increasing the at-home-duration, we have $h_{t-\tau} = 20$, and thus

$$\begin{aligned} &log(R_t) = log(1.4) - 0.0236 \cdot 20 \approx -0.13553 \\ &\Leftrightarrow R_t \approx 0.87 \\ &\Leftrightarrow \Delta R_t \approx 0.87 - 1.4 = -0.53 \end{aligned}$$

Again, this needs to be compared with our range of $[-0.75, -0.6]$.

The trend variable $days$ leads to even smaller reductions over time, but after a year, $days = 365$ even with $h_{t-\tau} = 0$ leads to an $R_t < 1$, which is clearly not plausible. Indeed, the model was estimated against data until June 23rd, 2020, and it is plausible that the trend term, at least in part, rather captures the seasonal effect.

Comparison with [22] For the "residential" category, [22] obtain the following model:

$$R_t = \beta_0 + \beta_r \cdot h_{t-\tau},$$

where h denotes the percentage change of the "residential" category. For the 14-day lag ($\tau = 14$) the coefficient differs between the considered five public health units in the Greater Toronto Area and takes on values between -0.035 and -0.018. Consequently, this leads to a reduction of the R-value between $-0.035 \cdot 20 = -0.7$ and $-0.018 \cdot 20 = -0.36$, which overlaps with our range of $[-0.75, -0.6]$.
